# Secular trends of morbidity and mortality of prostate, bladder, and kidney cancers in China, 1990 to 2019 and their predictions to 2030

**DOI:** 10.1186/s12885-022-10244-9

**Published:** 2022-11-11

**Authors:** Qiao Huang, Hao Zi, Lisha Luo, Xuhui Li, Cong Zhu, Xiantao Zeng

**Affiliations:** 1 Center for Evidence-Based and Translational Medicine, Zhongnan Hospital of Wuhan University, #169, East Lake Road, Wuchang District, Hubei Province Wuhan City, China; 2 Department of Evidence-Based Medicine and Clinical Epidemiology, Second School of Clinical Medicine, Wuhan University, Wuhan, China; 3 Department of Urology, Zhongnan Hospital of Wuhan University, Wuhan, China

**Keywords:** Genitourinary cancer, Prostate, Bladder, Kidney, Morbidity, Mortality, Prediction

## Abstract

**Background:**

Prostate, bladder and kidney cancers are common age-related genitourinary cancers. China's population is aging at an increasing rate, so predicting the morbidity and mortality of prostate, bladder, and kidney cancer in China is of great significance to provide epidemiological evidence for forward planning and implementation of national health policies.

**Methods:**

Numbers of incidences and deaths by cancer (prostate, bladder and kidney), sex (male and female) and age groups from 1990 to 2019 were extracted from the Global Burden of Disease (GBD) Study. We applied Bayesian age-period-cohort models to predict incidences and deaths to 2030. We also calculated Age-standardized incidence rate (ASIR) and mortality rate (ASMR), their trends were quantified by estimated average percentage change (EAPC) and 95% confidence interval (CI).

**Results:**

Predictions suggest that by 2030, there will be 315,310 prostate cancer cases, 192,390 bladder cancer cases and 126,980 kidney cancer cases. The ASIRs will increase to 25.54/100,000 for prostate cancer (EAPC: 2.88, 95% CI, 2.84, 2.93), 7.54/100,000 for bladder cancer (EAPC: 2.58, 95% CI, 2.54, 2.61) and 5.63/100,000 for kidney cancer (EAPC: 4.78, 95% CI, 4.54, 5.02). Number of deaths in 2030 will be 81,540, 61,220, and 41,940, respectively. Different ASMR changes are observed, the ASMR for prostate cancer will drop to 7.69/100,000 (EAPC: -0.29, 95% CI, -0.31, -0.27), the ASMR for bladder cancer will stabilize at 2.49/100,000 (EAPC: 0.00, 95% CI, -0.02, 0.03), the ASMR of kidney cancer will increase to 1.84/100,000 (EAPC: 3.45, 95% CI, 3.22, 3.67). From 1990 to 2030, higher numbers of cases and rates are reported among males and in the 60 plus age group, both ASIR and ASMR of bladder and kidney cancers presents progressively widening differences between both males and females and between the < 60 and the ≥ 60 age groups.

**Conclusion:**

Morbidity and mortality of the three genitourinary cancers are predicted to increase further over the next decade. It highlights the need for timely development and implementation of optimal health policies to curb the epidemic trends.

**Supplementary Information:**

The online version contains supplementary material available at 10.1186/s12885-022-10244-9.

## Introduction

Cancer has been a leading global public health problem, both cancer incidence and mortality continue to increase [1]. Genitourinary cancer is a type of malignancy involving the urinary system, including common prostate cancer, bladder cancer and kidney cancer. Obtaining accurate information and evaluating trends of disease burden of the three genitourinary cancers were of great significance, as this will help countries initialize optimal cancer control strategies. In 2019, the Global Burden of Diseases, Injuries, and Risk Factors Study (GBD) showed that the global incidence of cases reached 524,300 for bladder cancer, 371,750 for kidney cancer, and 1410,450 for prostate cancer, which contributed to 228,790 deaths, 166,440 deaths and 486,840 deaths, respectively [2]. Both incidence rates and mortality rates of the three cancers increased with age, especially in the above 50 age group with males having a higher incidence and mortality than females [2–4].

China remains the country with the largest population in the world. Based on China’s seventh national population census 2020, its population reached about 1.4 billion (51.24% males and 48.76% females) accounting for about 18% of the world's total population. According to the 2019 revision of world population prospects, China's population will peak at 1.5 billion in 2030 [5]. However, China's demographic history shows that China's population is aging rapidly [6]. The proportion of people over 65 years old was 8.9% in 2010 and had increased to 13.5% by 2020. By 2030, it will rise to 18.21% [7]. This rapid aging will present China with many challenges. The predominant influence might be an inevitable increase in the population disease burden. Disease burden is measured to quantify the impact of health problem. Prolonged human lifespan and aging in China will increase the burden of age-related non-communicable diseases [8]. In 2019, China had the highest incidence cases of bladder cancer (100,020.20), the most deaths from prostate cancer (54,390.88), bladder cancer (40,094.24) and kidney cancer (23,954.24) among 204 countries and territories [2].

With the population growth and rapid aging process in China, the three genitourinary cancers will keep increasing which can impose a considerable burden on the whole society. This should focus our attention on taking early action. Projection of the three genitourinary cancers will provide valuable information for adjustment of health policies and optimal allocation of medical resources. In this study, we extracted data from the GBD 2019 database and implemented a Bayesian age-period-cohort (BAPC) model to project incidence and mortality of the three cancers up to 2030.

## Materials and methods

### Data source

The GBD database covers morbidity and mortality data for more than 350 diseases in approximately 204 countries from 1990 to 2019. Details about metrics and estimations from the GBD database have been published in a previous study [9]. In this study, we extracted numbers of incidences and deaths for China by cancer type (prostate, bladder and kidney), sex (male and female) and age groups (21 groups: < 1 years, from 1 ~ 4 years to 90 ~ 94 years at 5 year intervals, and > 95 years). The online Global Health Data Exchange query tool was used to extract data. To project the incidence and mortality by 2030, we downloaded the corresponding population data from the Department of Economic and Social Affairs (United Stations). In the World Population Prospects 2019, population in China has been projected until 2050. Population data was organized by sex and the same 21 age groups. All data extraction was completed before April 1, 2021. Summary information from a public database was analyzed, so ethical approval could be exempted.

### Statistical analysis

#### Selection of projection model

Several methods have been developed to predict cancer incidence and death using cancer registry data, including age-period-cohort (APC) model (Poisson distribution), Bayesian age-period-cohort (BAPC) model, Poisson regression (population size as offset), negative binomial regression (population size as offset), generalized additive model (Poisson distribution and restricted maximum likelihood estimation), and Nordpred model. The available Nordpred package in R program requires 18 fixed age groups and cannot predict more than 5 periods [10]. However, available data for the three cancers had different numbers of age groups in terms of incidence and mortality, so the Nordpred model was excluded. We selected the best model for predicting the three cancers by comparing the predictive performance of these models, details of model parameters are available in the Supplement 1 (R codes for model comparison).

For mortality and morbidity, numbers from the total population, male population and female population were split into training sets (from 1990 to 2014) and testing sets (2015 to 2019), respectively. Prediction accuracy was assessed using the mean absolute percentage error (MAPE), calculated by $\frac{1}{n}\times \sum_{i}^{n}\frac{|\widehat{{Y}_{i}}-{Y}_{i}|}{{Y}_{i}}$, where Y denoted observed case, $\widehat{Y}$ denoted predicted case, *i* denoted the predicted *i*^*th*^ year and n denoted total number of years with prediction. The MAPEs in total, male and female population were summarized for the 5 selected models (Supplement 2 table). Overall error rate of the BAPC model was relatively lower than other 4 models. Meanwhile, previous comparative study have showed that BAPC model gave well calibrated projection and not too wide uncertainty interval [11]. Finally, the BAPC model was used for projection of the three cancers up to 2030.

#### Projection of incidence case and death cases for bladder, kidney and prostate cancer

Both cases (incidence or mortality) and population were collected for each 5-year age group and 1-year period from 1990 to 2019, so the grid factor was set as 5. Integrated nested Laplace approximations (INLA, http://www.r-inla.org) and full Bayesian inference were integrated in the BAPC model. An embedded Poisson model was fitted for projecting both the incidence and deaths.

Age, period and cohort effects can be modeled using either random walk of either first-order (RW1, fixed effect) or second order (RW2, linear effect). Previous review have demonstrated that RW2 priors gave more reliable forecasts than RW1 priors [12]. We assumed independent mean-zero normal distributions by restricting second-order differences towards zero for all effects and specified the RW2. Age, period and cohort effects shared the same log-gamma prior distribution for the precision parameter. After the BAPC model was fitted, parameter variances were summarized for checking. Median, 2.5% quantile and 97.5% quantile of counts were projected.

#### Crude rate and age-standardized rate

Crude rate was calculated by a ratio of the number of new cases (morbidity) or deaths (mortality) to the number of population at risk. To simplify demonstration, the 21 age groups were combined into 5 groups (0 ~ 19 years, 20 ~ 39 years, 40 ~ 59 years, 60 ~ 79 years and 80 ~ years) and corresponding crude rates were calculated separately. Age distribution might change over time and size of populations varies in different geographical areas, age-standardized rate (ASR) was estimated for comparison. The ASR is a weighted mean of age-specified rates per 100, 000 people, where the weights are age-specified proportions in a standard population. We selected the GBD world population 2019 as the reference population to calculate age standardized incidence rate (ASIR) and age standardized mortality rate (ASMR). Meanwhile, raw age-standardized rates were compared with predicted age-standardized rates.

#### Temporal trend estimation

Incidence rate and mortality rate were summarized from 1990 to 2019 and were projected until 2030. To reflect temporal trend of both rates, we calculated the estimated average percentage change (EAPC) which is a commonly used indicator for quantifying trend. Generalized linear models based on Gaussian distribution were fitted, namely *ln (rate)* = *γ* + *β ⋅ t* + *ε*. The EAPC and its 95% confidence interval (CI) can be calculated using 100 × (${\mathrm{e}}^{\upbeta }$-1). In this study, the EAPCs were calculated for the 1990 ~ 2019 interval and 2020 ~ 2030 interval, respectively. If the upper boundary of the 95% CI was less than 0, it suggested rate was decreasing over time. If the lower boundary of the 95% CI was greater than 0, it suggested rate was increasing over time. The rate was considered stable if the 95% CI included 0.

All statistical analyses and data visualization were performed using the R program (version 4.0.3, R core team, Vienna, Austria) with apc package (APC model), bapc package (BAPC model), nordpred package (Nordpred model), stats package (Poisson regression), MASS package (negative binomial regression), mgcv package (generalized additive model) and ggplot2 package (data visualization). A two-sided *P* value < 0.05 was considered as statistical significance.

## Results

### Prostate cancer

#### Incidence and Mortality in 2019 and temporal trend from 1990 to 2030

Counts of prostate cancer in 2019 were 153,450 with an ASIR of 18.72/100,000, which contributed to 54,390 deaths with an ASMR of 8.09/100,000 (Tables 1 and 2). The majority of cases and deaths occurred in ≥ 60 age groups. The ASIRs were 100.44/100,000 in the 60 ~ 79 age group and 289.91/100,000 in the 80 ~ age group, corresponding ASMRs were 28.15/100,000 and 215.15/100,000. The peaks of age-specified incidences and deaths were in the 70 ~ 74 age group and 80–84 age group, respectively. In > 45 age groups, both morbidity and mortality rates can be seen to increase with age (Fig. S1).

**Table 1:** Number and incidence rate of bladder, kidney and prostate cancer by sex and age at 1990, 2019 and projected 2030

		Number of cases (95% UI) (per 1000)			Incidence rate (95% UI) (× 100,000)			EAPC (95% CI)	
		1990	2019	2030	1990	2019	2030	1990 ~ 2019	2020 ~ 2030
Prostate	Male ^a^	26·44(19·45,33·23)	153·45(115·66,209·60)	315·31(223·82,406·79)	8·69(6·53,11·15)	18·72(14·18,25·27)	25·54(18·11,32·97)	3·00(2·86,3·15)	2·88(2·84,2·93)
	Age group								
	0 ~ 19 ^b^	-	-	-	-	-	-	-	-
	20 ~ 39 ^b^	0·35(0·24,0·44)	1·07(0·83,1·46)	1·15(0·63,1·68)	0·17(0·11,0·21)	0·49(0·38,0·67)	0·62(0·34,0·91)	3·92(3·76,4·09)	2·44(2·10,2·78)
	40 ~ 59 ^b^	3·06(2·04,3·96)	16·41(11·75,23·47)	23·97(16·98,30·96)	2·92(1·95,3·77)	7·39(5·30,10·58)	10·76(7·62,13·90)	3·58(3·34,3·81)	2·77(2·12,3·41)
	60 ~ 79 ^b^	18·58(13·63,22·89)	106·44(80·11,146·99)	225·10(159·97,290·22)	40·57(29·77,50·00)	100·44(75·59,138·70)	143·66(102·10,185·22)	3·41(3·23,3·60)	3·30(3·19,3·41)
	80 ~ ^b^	4·45(3·54,5·94)	29·53(22·97,37·69)	65·09(46·25,83·94)	165·54(131·57,220·90)	289·91(225·55,370·07)	398·53(283·16,513·91)	2·46(2·14,2·79)	3·24(3·02,3·46)
Bladder	Total ^a^	25·55(21·85,29·58)	100·02(81·83,120·39)	192·39(118·60,266·17)	3·38(2·89,3·90)	5·59(4·60,6·69)	7·54(4·64,10·44)	2·04(1·91,2·17)	2·58(2·54,2·61)
	Male ^a^	18·36(21·85,29·58)	82·68(81·83,120·39)	160·04(99·09,220·99)	5·38(6·62,8·92)	9·92(10·05,14·53)	13·46(8·32,18·60)	2·46(2·31,2·60)	2·66(2·62,2·69)
	Female ^a^	7·19(21·85,29·58)	17·34(81·83,120·39)	30·36(17·09,43·63)	1·82(5·36,7·24)	1·87(8·66,12·64)	2·28(1·27,3·29)	0·16(0·05,0·27)	1·80(1·77,1·83)
	Age group								
	0 ~ 19 ^b^	0·22(0·18,0·27)	0·17(0·14,0·21)	0·23(0·02,0·44)	0·18(0·15,0·21)	0·21(0·17,0·26)	0·27(0·03,0·51)	0·24(0·03,0·44)	2·80(2·63,2·96)
	20 ~ 39 ^b^	2·25(1·91,2·65)	4·58(3·77,5·48)	4·02(2·40,5·65)	0·54(0·46,0·64)	1·10(0·91,1·31)	1·16(0·69,1·63)	2·24(2·08,2·40)	1·48(0·90,2·07)
	40 ~ 59 ^b^	6·20(5·22,7·25)	22·36(17·57,27·74)	30·50(18·82,42·18)	3·09(2·60,3·61)	5·12(4·02,6·35)	7·00(4·32,9·68)	2·14(1·92,2·36)	2·15(1·78,2·53)
	60 ~ 79 ^b^	13·58(11·71,15·65)	55·17(45·18,66·59)	119·79(74·00,165·57)	14·69(12·66,16·93)	25·48(20·87,30·75)	37·17(22·96,51·37)	2·15(1·94,2·37)	3·10(2·98,3·22)
	80 ~ ^b^	3·29(2·83,3·76)	17·74(15·16,20·37)	37·85(23·37,52·33)	43·94(37·71,50·22)	69·23(59·17,79·52)	91·75(56·64,126·86)	2·03(1·71,2·35)	2·78(2·65,2·91)
Kidney	Total ^a^	11·07(9·30,13·08)	59·83(48·82,72·66)	126·98(12·62,241·71)	1·16(0·98,1·36)	3·34(2·73,4·04)	5·63(0·53,10·79)	4·59(4·10,5·07)	4·78(4·54,5·02)
	Male ^a^	6·16(4·76,7·79)	42·55(32·52,54·27)	102·91(0·00,218·36)	1·32(1·03,1·66)	4·79(3·69,6·07)	9·09(0·00,19·34)	5·59(5·04,6·15)	5·77(5·45,6·10)
	Female ^a^	4·92(3·94,6·03)	17·27(13·44,21·87)	30·96(9·53,52·42)	1·02(0·82,1·24)	1·98(1·54,2·51)	2·91(0·80,5·02)	2·88(2·50,3·26)	3·56(3·39,3·72)
	Age group								
	0 ~ 19 ^b^	2·58(2·12,3·07)	2·51(2·06,3·03)	2·60(0·06,5·50)	0·56(0·46,0·66)	0·74(0·61,0·89)	0·90(0·02,1·90)	1·52(1·18,1·86)	1·96(1·43,2·49)
	20 ~ 39 ^b^	1·42(1·18,1·69)	6·30(5·19,7·58)	7·02(0·70,13·35)	0·34(0·28,0·41)	1·51(1·25,1·82)	2·02(0·20,3·84)	6·40(5·72,7·08)	2·88(2·25,3·51)
	40 ~ 59 ^b^	3·18(2·66,3·83)	22·44(17·81,28·04)	44·60(4·49,84·72)	1·59(1·33,1·91)	5·14(4·08,6·42)	10·23(1·03,19·43)	5·29(4·71,5·88)	5·52(5·41,5·63)
	60 ~ 79 ^b^	3·45(2·97,3·99)	23·77(19·72,28·42)	61·40(6·19,116·61)	3·73(3·21,4·31)	10·98(9·11,13·12)	19·05(1·92,36·18)	4·50(3·97,5·03)	5·39(5·17,5·61)
	80 ~ ^b^	0·45(0·38,0·51)	4·80(4·04,5·59)	11·37(1·14,21·60)	5·97(5·11,6·79)	18·75(15·76,21·83)	27·56(2·76,52·37)	5·48(4·84,6·12)	3·59(3·18,4·01)

*EAPC* estimated annual percentage change, *95% UI* 95% uncertainty interval, *95% CI* 95% confidence interval

^a^ age-standardized incidence rate

^b^ crude incidence rate in each age group

**Table 2:** Number and mortality rate of bladder cancer, kidney cancer and prostate cancer by sex and age at 1990, 2019 and projected 2030

		Number of deaths (95% UI) (per 1000)			Mortality rate (95% UI) (per 100,000)			EAPC (95% CI)	
		1990	2019	2030	1990	2019	2030	1990 ~ 2019	2020 ~ 2030
Prostate	Male ^a^	20·38(15·08,25·72)	54·39(41·82,72·32)	81·54(61·83,101·25)	7·96(6·03,10·36)	8·09(6·23,10·57)	7·69(5·83,9·56)	0·11(0·04,0·18)	-0·29(-0·31,-0·27)
	Age group								
	0 ~ 19 ^b^	-	-	-	-	-	-	-	-
	20 ~ 39 ^b^	0·16(0·11,0·20)	0·14(0·11,0·19)	0·11(0·05,0·17)	0·08(0·05,0·09)	0·07(0·05,0·09)	0·06(0·03,0·09)	-0·88(-1·14,-0·62)	-0·68(-1·01,-0·35)
	40 ~ 59 ^b^	1·56(1·04,2·00)	2·50(1·81,3·54)	2·57(1·91,3·23)	1·49(0·99,1·91)	1·13(0·82,1·59)	1·15(0·86,1·45)	-1·11(-1·25,-0·97)	-0·42(-1·14,0·30)
	60 ~ 79 ^b^	13·51(9·89,16·59)	29·83(22·77,40·94)	45·32(34·41,56·23)	29·51(21·59,36·22)	28·15(21·49,38·63)	28·92(21·96,35·89)	-0·23(-0·45,-0·01)	0·15(0·02,0·29)
	80 ~ ^b^	5·15(4·04,6·94)	21·91(17·12,27·65)	33·54(25·47,41·62)	191·55(150·39,258·18)	215·15(168·14,271·47)	205·37(155·92,254·83)	0·70(0·47,0·94)	0·09(0·06,0·13)
Bladder	Total ^a^	17·29(14·81,19·85)	40·09(33·55,47·67)	61·22(43·37,79·09)	2·65(2·27,3·04)	2·49(2·09,2·94)	2·49(1·76,3·21)	-0·13(-0·20,-0·06)	0·00(-0·02,0·03)
	Male ^a^	11·91(9·81,14·09)	31·50(25·37,38·53)	47·53(34·02,61·03)	4·30(3·57,5·05)	4·43(3·62,5·35)	4·37(3·13,5·62)	0·22(0·12,0·32)	0·00(-0·01,0·02)
	Female ^a^	5·38(4·37,6·57)	8·59(6·74,10·62)	13·42(7·92,18·92)	1·53(1·25,1·86)	0·99(0·78,1·22)	1·00(0·59,1·41)	-1·53(-1·62,-1·44)	0·07(0·05,0·09)
	Age group	-	-	-	-	-	-	-	-
	0 ~ 19 ^b^	0·05(0·04,0·06)	0·01(0·01,0·02)	0·01(0·00,0·03)	0·04(0·04,0·05)	0·02(0·01,0·02)	0·01(0·00,0·03)	-3·75(-4·03,-3·47)	-0·72(-0·93,-0·52)
	20 ~ 39 ^b^	0·67(0·56,0·78)	0·46(0·38,0·55)	0·27(0·16,0·38)	0·16(0·14,0·19)	0·11(0·09,0·13)	0·08(0·04,0·11)	-1·95(-2·23,-1·68)	-2·11(-2·69,-1·52)
	40 ~ 59 ^b^	3·04(2·55,3·56)	4·66(3·74,5·78)	4·18(2·94,5·43)	1·52(1·27,1·77)	1·07(0·86,1·32)	0·96(0·67,1·25)	-1·15(-1·29,-1·01)	-1·43(-1·94,-0·92)
	60 ~ 79 ^b^	9·88(8·51,11·31)	21·20(17·60,25·50)	34·11(24·21,44·01)	10·68(9·20,12·23)	9·79(8·13,11·78)	10·58(7·51,13·66)	-0·29(-0·53,-0·05)	0·48(0·28,0·68)
	80 ~ ^b^	3·65(3·15,4·14)	13·77(11·81,15·83)	22·65(16·07,29·24)	48·75(42·02,55·32)	53·73(46·10,61·78)	54·91(38·95,70·88)	0·62(0·36,0·87)	0·54(0·48,0·60)
Kidney	Total ^a^	5·88(4·94,6·91)	23·95(19·64,28·82)	41·94(0·00,91·21)	0·70(0·59,0·81)	1·34(1·10,1·60)	1·84(0·00,4·03)	3·06(2·62,3·50)	3·45(3·22,3·67)
	Male ^a^	3·33(2·61,4·18)	16·88(12·99,21·23)	31·67(0·00,74·43)	0·82(0·65,1·01)	1·98(1·54,2·47)	2·90(0·00,6·84)	4·06(3·57,4·56)	3·97(3·70,4·25)
	Female ^a^	2·55(2·07,3·08)	7·07(5·54,8·81)	10·93(0·92,21·06)	0·60(0·49,0·72)	0·77(0·60,0·96)	0·93(0·07,1·83)	1·35(1·01,1·70)	2·23(2·09,2·36)
	Age group								
	0 ~ 19 ^b^	0·62(0·51,0·75)	0·37(0·30,0·44)	0·31(0·00,0·92)	0·14(0·11,0·16)	0·11(0·09,0·13)	0·11(0·00,0·32)	-0·29(-0·62,0·03)	0·44(-0·40,1·30)
	20 ~ 39 ^b^	0·61(0·50,0·72)	1·59(1·29,1·94)	1·95(0·00,4·24)	0·15(0·12,0·17)	0·38(0·31,0·46)	0·56(0·00,1·22)	4·35(3·70,5·01)	3·95(3·74,4·15)
	40 ~ 59 ^b^	2·06(1·71,2·47)	8·28(6·55,10·27)	14·74(0·00,31·97)	1·03(0·85,1·23)	1·90(1·50,2·35)	3·38(0·00,7·33)	2·97(2·59,3·36)	4·60(4·50,4·71)
	60 ~ 79 ^b^	2·08(1·79,2·39)	9·32(7·78,11·04)	18·02(0·00,39·08)	2·25(1·94,2·59)	4·30(3·59,5·10)	5·59(0·00,12·12)	2·74(2·17,3·32)	2·66(2·40,2·92)
	80 ~ ^b^	0·50(0·43,0·57)	4·40(3·71,5·14)	6·92(0·00,15·01)	6·74(5·75,7·64)	17·19(14·50,20·05)	16·77(0·00,36·39)	4·59(4·00,5·18)	1·95(1·85,2·06)

*EAPC* estimated annual percentage change, *95% UI* 95% uncertainty interval, *95% CI* 95% confidence interval

^a^ age-standardized mortality rate

^b^ crude incidence rate in each age group

#### Projections of incidence and mortality from 2020 to 2030

**Fig. 1:**
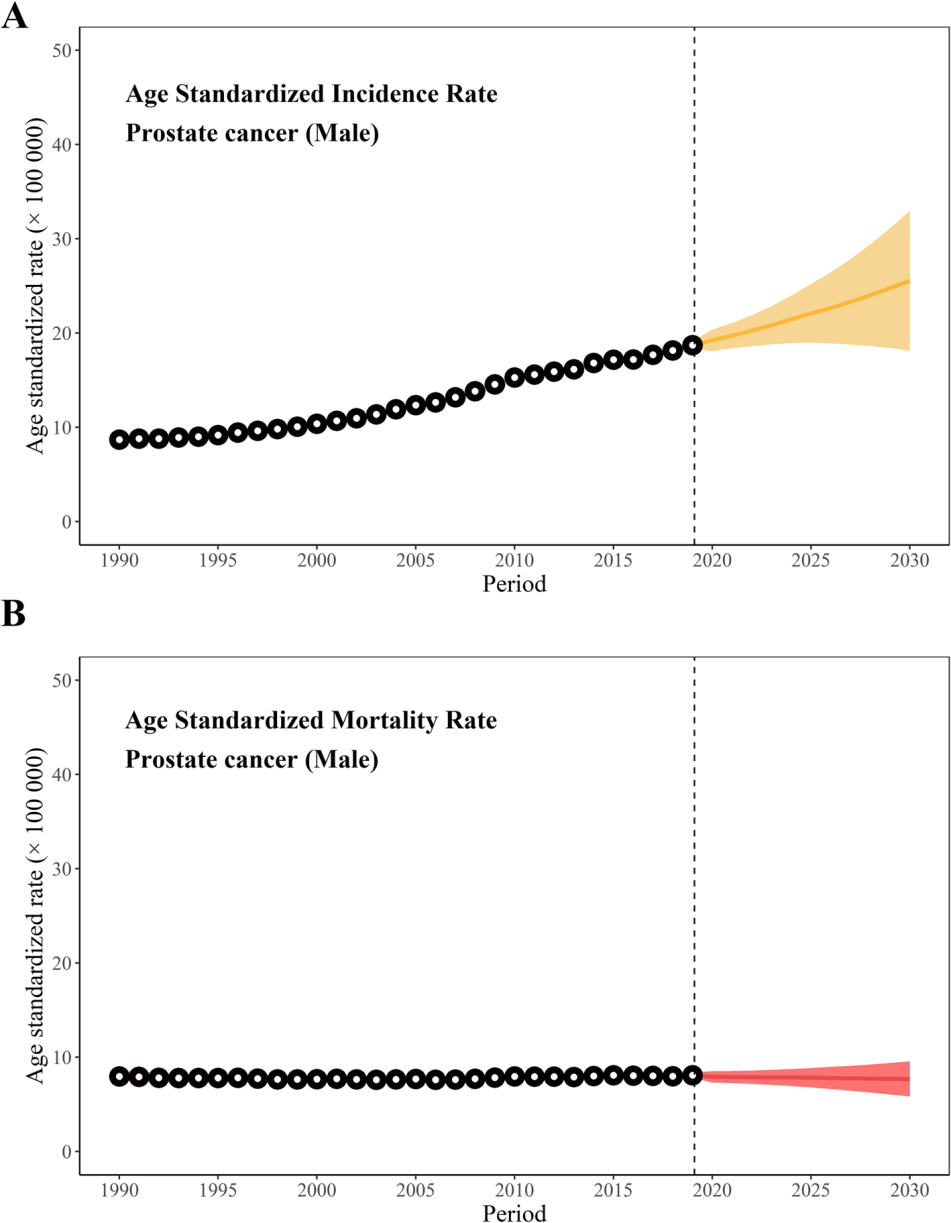
Age standardized incidence rate and mortality rate of prostate cancer from 1990 to 2030

The projected number of new cases in 2030 will be 315,310 (Table 1, Fig. S2-A). Its ASIR shows an unfavorable increase with a significant EAPC of 2.88 (95%CI, 2.84, 2.93) (Table 1, Fig. 1-A). Increases in incidence and crude incidence rate will be observed in 4 age groups (20 ~ 39, 40 ~ 59, 60 ~ 79, 80 ~), especially in the 60 ~ 79 age group with the highest EPAC of 3.30 (95% CI, 3.19, 3.41).

There will be 81,540 deaths from prostate cancer in 2030 (Table 2, Fig. S2-B). The ASMR will slightly decrease from 8.09/100,000 in 2019 to 7.69/100,000 in 2030 with a significant EAPC of -0.29 (95% CI, -0.31, -0.27) (Table 2, Fig. 1-B). The number of deaths shows an increasing trend in the 60 ~ 79 age group and the 80 plus age group, the crude mortality rate will remain nearly constant (Table 2).

## Bladder cancer

### Incidence and mortality in 2019

In 2019, the numbers of new bladder cancers were 100,020 in the total population, 82,680 in males group and 17,340 in females group, corresponding ASIR were 5.59/100,000, 9.92/100,000 and 1.87/100,000, respectively (Table 1). 40,900 deaths were observed in total population with an ASMR of 2.49/100,000, including 31,500 in males and 8,590 in females (Table 2). Numbers of cases and deaths in the 60 ~ 79 age group were 55,170 and 21,200, both of which accounted for about a half. The crude rate in 80 plus age group was as high as 69.23/10000 for incidence and 53.73/100,000 for mortality (Tables 1 and 2). Among males and females, the highest age-specified cases and deaths were observed in the 70 ~ 74 and 80 ~ 84 age groups, respectively. Both crude incidence rate and crude mortality rate increased with age and peaked in the 95 + age group. In all age groups, the number of cases, deaths, crude incidence rate and mortality rate in the male group were consistently higher than those in the female group (Fig. S3).

### Projections of incidence and mortality from 2020 to 2030

**Fig. 2:**
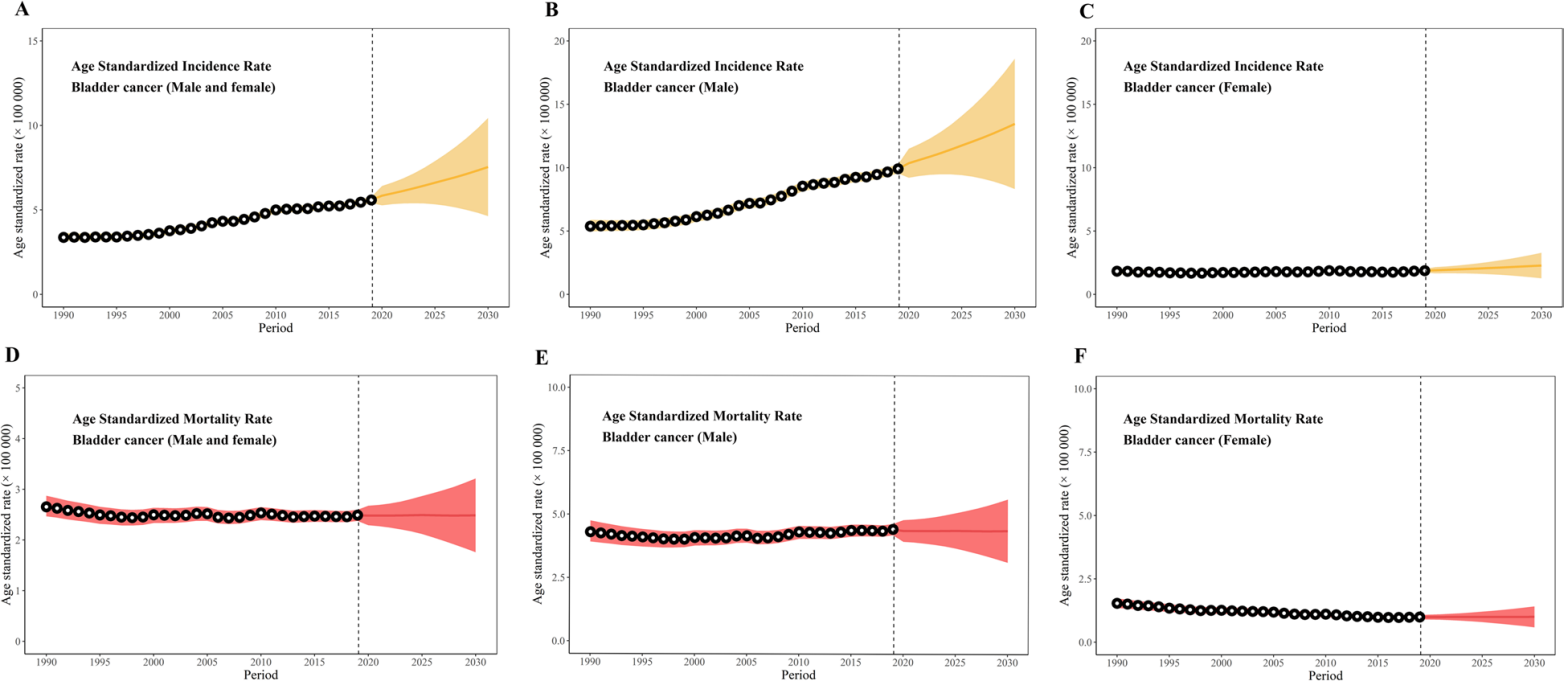
Age standardized incidence rate and mortality rate of bladder cancer from 1990 to 2030

By 2030, there will be 192,390 new cases of bladder cancer in China, and the ASIR will increase to 7.54/100,000 with an EAPC of 2.58 (95%CI, 2.54, 2.61) (Table 1, Fig. 2-A and Fig. S4-A). Numbers of new cases in both males and females will increase by about 2 times and are expected to be 160,040 and 30,360, with ASIRs of 13.46/100,000 and 1.87/100,000, respectively. The increasing speed of rise in ASIR in males with an EAPC of 2.66 (95% CI, 2.62, 2.69) will be higher than in females with an EAPC of 1.80 (95%CI, 1.77, 1.83). (Table 1, Fig. 2-B, C and Fig. S4-B, C). Number of new cases and incidence rate will remain stable in the 0 ~ 59 age group, but show a great increase in both 60 ~ 79 age group and 80 plus age group. The crude incidence rate will reach 37.17/100,000 in the 60 ~ 79 age group with an EAPC of 3.10 (95% CI, 2.98, 3.22) and 71.75/100,000 in the 80 plus age group with an EAPC of 2.78 (95% CI, 2.65, 2.91) (Table 1).

**Fig. 3:**
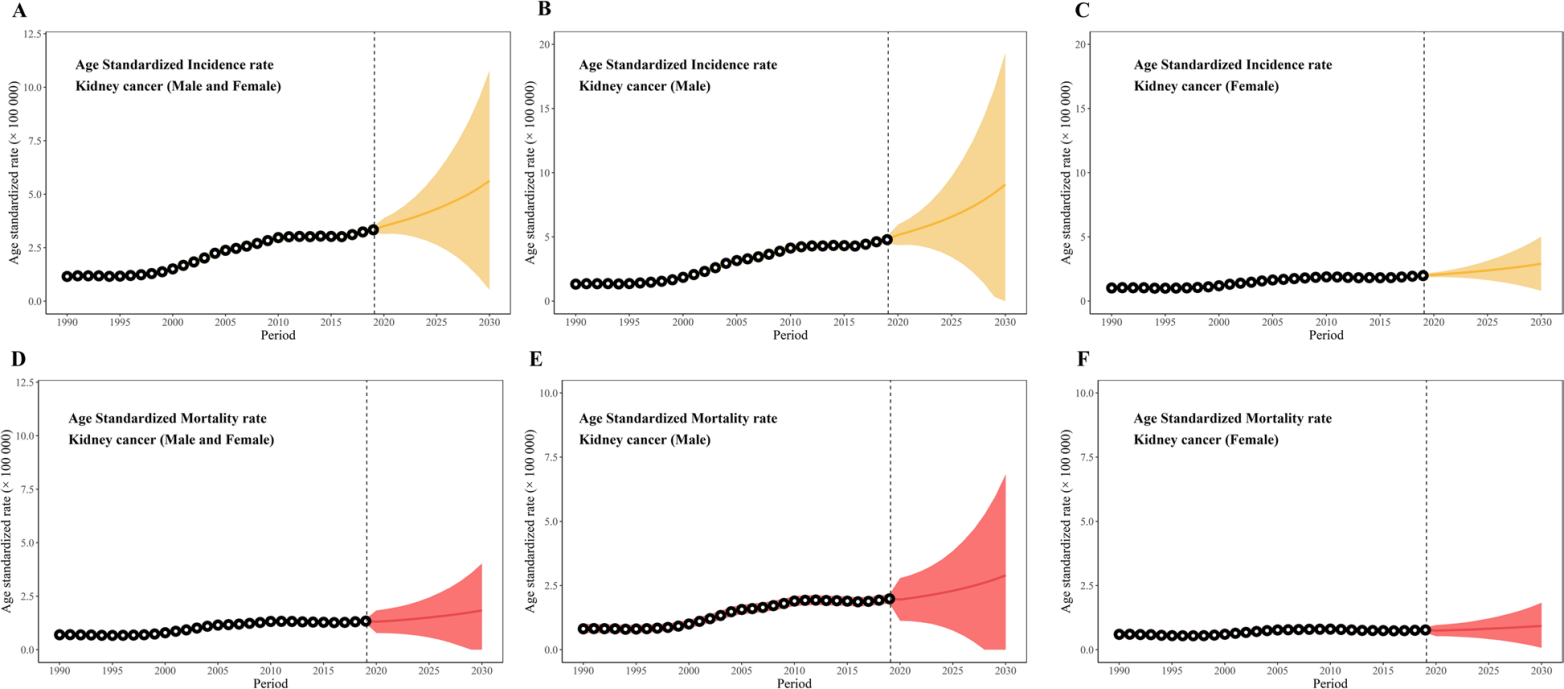
Age standardized incidence rate and mortality rate of kidney cancer from 1990 to 2030

The number of deaths for bladder cancer will increase slightly to 61,220, the corresponding ASMR will remain stable at 2.49/100,000 with a non-significant EAPC of 0.00 (95%CI, -0.02, 0.03) (Table 2, Fig. 2-D and Fig. S4-D). Similar changes (slight increases of deaths and stable ASMR) will be observed in both males and females (Table 2, Fig. 3 E, F and Fig. S4-D, F). But, age-specified deaths and mortality rates show a different pattern of change. Number of deaths and ASMRs will decrease in the 0 ~ 19, 20 ~ 39 and 40 ~ 59 groups with significant and negative EAPC, but increase in both the 60 ~ 79 and 80 plus age groups with significant and positive EAPC (Table 2).

## Kidney cancer

### Incidence and mortality in 2019 and temporal trend from 1990 to 2019

For kidney cancer, 59,830 new cases were reported, including 42,550 in males and 17,270 in females. It contributed to ASIRs of 3.34, 4.79 and 1.98 per 100,000 people in the total population, males and females (Table 1). The numbers of deaths were 23,950 in total population, 16,880 in males and 7,070 in females with ASMRs of 1.34, 1.98 and 0.77 per 100,000 people, respectively. About three-quarters of incidences and deaths occurred in the 40 ~ 79 age group (Table 2). Similar results were observed in both males and females (Fig. 2). In both males and females before 14 years, the crude incidence rates decreased and the crude mortality rates were close to 0. After the 20 years age group, both crude incidence rate and mortality rate increased by age and peaked at the 95 + age group, and males showed a higher rate than females (Fig. S5).

### Projections of incidence and mortality from 2020 to 2030

In 2030, number of new cases for kidney cancer will expand to be 126,980, corresponding ASMR will be elevated to 5.63/100,000 with a significant EAPC of 4.78 (95%CI, 4.54 to 5.02) (Table 1, Fig. 3-A and Fig. S6-A). The double growth of incidence and ASIR will also be observed in both males and females, 102,910 and 30,960 for new cases, 9.90/100,000 and 2.91/100,000 for ASIRs, 4.78 (95%CI, 4.54,5.02) and 5.77 (95%CI, 5.45,6.10) for EAPCs, respectively (Table 1, Fig. 3-B, C and Fig. S6-B, C). Age-specified numbers of incidence and crude incidence rate will increase slightly in both 0 ~ 19 age group and 20 ~ 39 age group, however, they will be doubled in all > 40 age groups with high EAPC, 5.52 (95% CI, 5.41, 5.63) for the 40 ~ 59 age group, 5.39 (95% CI, 5.17, 5.61) for the 60 ~ 79 age group, 3.59 (95% CI, 3.18, 4.01) for the 80 plus age group. The highest age-specified incidence rate will be 27.56/100,000 in 80 plus age group (Table 1).

The number of deaths from kidney cancer will approximately double from 23,950 to 41,940 in the total population, from 16,880 to 31,670 in males, and 7,070 to 10,930 in females. Corresponding ASMRs will reach 1.84/100,000 (EAPC = 3.45, 95% CI, 3.22, 3.67), 2.90/100,000 (EAPC = 3.97, 95% CI, 3.70, 4.25) and 0.93/100,000 (EAPC = 2.23, 95% CI, 2.09, 2.36), respectively (Table 2, Fig. 3-D, E, F and Fig. S6-D, E, F). The majority of deaths will still occur in the 40 ~ 79 age group, with 14,740 deaths in the 40 ~ 59 age group and 18,020 deaths in the 60 ~ 79 age group. The 40 ~ 59 age group will present the sharpest increase in crude mortality rate with an EAPC of 4.60 (95% CI, 4.50, 4.71) (Table 2).

## Discussion

China’s population continues to grow and is aging rapidly [13]. In the past 30 years, both morbidity and mortality rates of the genitourinary cancers in China have increased significantly [2]. In this study, we have applied a BAPC model with integrated nested Laplace approximations to project the mortality and morbidity of the three cancers for the next decade. By 2030, the ASIRs will increase to 25.54/100,000 for prostate cancer, 7.54/100,000 for bladder cancer and 5.63/100,000 for kidney cancer, respectively. Meanwhile, the ASMR will decrease to 7.69/100,000 for prostate cancer, remian stable at 2.49/100,000 for bladder cancer and increase to 1.84/100,000 for kidney cancer. This study reported a significant upward trend in ASIR with a stable ASMR. The disease burden caused by the three genitourinary cancers will further increase which will hinder harmonious and healthy development in China.

During the past 30 years, global downward trends in ASIRs were observed for prostate cancer and bladder cancer [2]. However, our study showed increasing trends of ASIRs in China from 1990 to 2030. Elevated ASIRs for kidney cancer have been reported both globally and in China. A previous global study predicted that the incidence rate of bladder cancer would substantially increase in middle SDI countries from 2017 to 2030, which is similar to our findings [14]. The increasing ASIRs might be mainly attributed to the increase in life expectancy and incident cases [15]. They might also be related to implementation of early disease screening in China, including routine prostate-specific antigen screening for prostate cancer [16], ultrasonography for kidney cancer [17], urine cytology and cystoscopy for bladder cancer [18]. With rapid social and economic development in China, increased exposure to risk factors, including a rising prevalence of smoking and Westernized lifestyle, have accelerated the epidemic of the three genitourinary cancers [19, 20]. There has been a substantial urban–rural disparity in the socioeconomic situation in China, which might bring about uneven distribution of lifestyle profiles. A cross-sectional study based on 36 cancer registers in China reported a higher cancer incidence in rural areas than in urban areas [21]. Chinese national preventive programs have provided early detection for population at high risk of multiple cancers, especially in rural area [22]. The cancer screening in high-risk individuals will further elevate the ASIRs of the three genitourinary cancers in the next decade.

The ASIRs for the three genitourinary cancers will rise. Analysis of cancer survival could provide alternative insights in evaluating the impact of cancers on the socioeconomic development of countries [23]. Our study shows that the three ASMRs in China were less variable from 1990 to 2030. China reported 3.04 million cancer deaths in 2020, the three genitourinary cancers accounted for about 3.89% [24]. By 2030, numbers of deaths will be as high as 81,540 cases for prostate cancer, 61,220 cases for bladder cancer, and 41,940 cases for kidney cancer. The ASMRs will be relatively stable, but the numbers of cancer deaths will increase. It is suggested that risk of mortality of the three cancers in China is mainly caused by aging. In the past 30 years, the global ASMR for bladder cancer and prostate cancer decreased [3]. The global death rate from bladder cancer was predicted to decrease in middle SDI countries from 2017 to 2030 [14]. However, the ASMR of bladder cancer in China was predicted to keep stable from 2020 to 2030. The ASMR of prostate cancer in China was also relatively stable, but it was always higher than bladder and kidney cancer. In China, prostate cancer has the seventh highest death rate among all cancers [25]. The highest ASMR among the three cancers demands increased great attention from decision-makers. Global ASMR of kidney cancer has reported an upward trend in the past 30 years [2]. In China, even though the ASMR of kidney cancer seems low, we should be aware that ASMR will increase from 1990 to 2030. Kidney cancer deaths attributable to high body mass index or high prevalence of obesity have been increasing considerably in China [26]. Effectively curbing obesity rates has reduced the mortality of kidney cancer in the United States [27]. Timely and national obesity prevention and management should be implemented in China to halt the alarming increase of obesity and ASMR for kidney cancer.

With development of cancer therapeutics, many novel therapeutic options are available to improve cancer patients’ survival rate [28, 29]. Recently developed poly ADP-ribose polymerase (PARP) inhibitors can be used for patients with metastatic castration-resistant prostate cancer [30]. Novel combination of immunotherapy with targeted drugs, including programmed death-1 (PD-1) inhibitor, cytotoxic T lymphocyte-associated antigen-4 (CTLA-4) inhibitor and PARP Inhibitors and immunotherapy, can improve response and reduce resistance for patients with bladder cancer [31]. A recent basic study showed novel histone methyltransferase EZH2 inhibitor can further reduce progression of urothelial carcinoma [32]. A translational study showed that combination therapy with the novel TKI sitravatinib and the anti–PD-1 immune checkpoint inhibitor improved the median progression-free survival for patients with kidney cancer [33]. Widespread availability of novel and favorable treatments can be appreciable at the population level to reduce the mortality rate of patients with all three cancers. However, the shortage of medical resources is a universal, long-term and global problem. Moreover, cancer health care has been frequently delayed and disrupted by the current COVID-19 pandemic [34]. Policy responses should guarantee cancer patients’ urgent access to medical resources, such as cancer diagnostic tests and cancer treatments.

According to the GBD 2019 database, the highest disability-adjusted life-years (DALY) had been reported for these three cancers in China [2]. Based on our prediction, the burden of the three genitourinary cancers will continue to increase in the next decade. It is crucial to get national support and planning and implement cross-sectoral cooperation and coordination among the government, public health organizations and individuals [35]. In 2016, the Healthy China 2030 blueprint was released at a national health conference, it sets a goal of increasing the proportion of the population with health literacy to 30% by 2030 [36]. National programs for the genitourinary cancers can include promoting a healthy lifestyle and reducing exposure to risk factors, developing national screening guidelines and increasing public willingness for screening, guaranteeing early detection, early diagnosis and early treatment in high-risk areas [37]. Moreover, technologies applicable to eligible cancers should be gradually incorporated into routine screening and treatment. These optimal strategies show promise in curbing the epidemic of the three genitourinary cancers in such a large country.

A high male–female ratio had been documented in China in the past two decades, the ratio has declined recently but still exceeds the normal level [38]. Meanwhile, China's aging population will continue to increase in the next decade [39]. Population aging could greatly influence cancer occurrence and cancer-related deaths [19]. Prostate, bladder, and kidney cancers were three men-favored and age-related cancers. In this study, great sex and age differences in morbidity and mortality of the three cancers were observed in China. From 1990 to 2030, ASIR and ASMR in males and the 60 plus age group are always higher, and the differences between males and female, and the difference between the < 60 age group and the ≥ 60 age group are gradually expanding. Ongoing monitoring of the differences in disease burden and establishing sex-aging-sensitive health policies would be needed to cope with their negative effects.

There are several limitations to this study. Firstly, the accuracy and robustness of the predictions depended on the accuracy of available morbidity and mortality data in the GBD database, progression in screening, diagnostic and treatment methods may have an effect on the morbidity and mortality, the predicted figures should be interpreted with cautions. Secondly, the COVID-19 pandemic is still ongoing and its impact is unprecedented. A significant drop in crude birth rates has been observed [40], which will consequently affect the population structure for several years. The contagious property of the COVID-19 virus will delay the diagnosis and treatment of cancer. This study didn’t account for impact of COVID-19 on cancer cases and deaths when we predicted morbidity and mortality for the next decade. Finally, we did not consider changes in risk factors of the three cancers, the predicted results might be interpreted with assumption of constant risk factor spectrum.

## Conclusions

Epidemiological data is imperative for planning future cancer prevention, screening, and treatment. In the next decade, incidence cases, mortality cases and ASIR of the prostate, bladder, kidney cancers are projected to increase significantly in China. Different change patterns but less variation in corresponding ASMRs are expected. The most pronounced increases are projected in males and the 60 plus age group. Our study has provided necessary evidence for assessment of health economics and better development of national health policies in advance to alleviate the disease burden imposed by prostate, bladder and kidney cancers in China.

## Supplementary Information

**Additional file 1.** R codes for comparing selected prediction models

**Additional file 2.** Mean absolute percentage error of 5 prediction models

**Additional file 3.** Figure S1 prostate cancer (1990-2019)

**Additional file 4.** Figure S2 prostate cancer, projection of cases and deaths to 2030

**Additional file 5.** Figure S3 bladder cancer (1990-2019)

**Additional file 6.** Figure S4 bladder cancer, projection of cases and deaths to 2030

**Additional file 7.** Figure S5 kidney cancer (1990-2019)

**Additional file 8.** Figure S4 kidney cancer, projection of cases and deaths to 2030

## Data Availability

The data used for projection are available in the Global Health Data Exchange query tool (https://ghdx.healthdata.org/gbd-2019).

## Abbreviations

ASIR: Age-standardized incidence rate
ASMR: Age-standardized mortality rate
APC: Age-period-cohort
BAPC: Bayesian age-period-cohort
CI: Confidence interval
EAPC: Estimated average percentage change
GBD: Global Burden of Diseases, Injuries, and Risk Factors Study
