# Supplementary material for: Secular trends of morbidity and mortality of prostate, bladder, and kidney cancers in China, 1990 to 2019 and their predictions to 2030
Source: BMC Cancer. 2022 Nov 11;22:1164. doi: 10.1186/s12885-022-10244-9 (PMC9650664; doi:10.1186/s12885-022-10244-9)
Supplement: Supplementary file 1 — Additional file 1. R codes for comparing selected prediction models [file 12885_2022_10244_MOESM1_ESM.pdf]

```r
#public function
assess.pred <- function(yhat,y){
  #Mean Absolute Percentage Error
  return(mean(abs(yhat-y)/y))
}

#-------------------------    prepare data -----------------------------------#
case <- DT_incidence[[1]]
pop  <- DT_incidence[[2]]
pop.all <- DT_incidence[[3]] #for nordpred

case <- DT_death[[1]]
pop  <- DT_death[[2]]
pop.all <- DT_death[[3]] #for nordpred
input.case <- case[1:25,] #1990-2014 for training
valid.case <- case[26:30,] #2015-2019 for validating
input.pop <-pop[1:nrow(input.case),]

numOFpred <- 5 #2015-2019
#-----------------------------------------------------------------------------#

#-------------------------  1 APC    --------------------------#
library(apc)
APC.data <- apc.data.list(response=as.matrix(input.case),data.format = 'PA',
                          age1 = 15,unit=5)
apc.fit.table(APC.data,"poisson.response") #choose APC model
APC.model <- apc.fit.model(APC.data,model.family = "poisson.response",
                           model.design='APC')
APC.pred <- apc.forecast.apc(APC.model)$trap.response.forecast
##get prediction with transpose
APC.pred <- t(APC.pred[,(nrow(input.case)+1):(nrow(input.case)+numOFpred)])
APC.accuracy <- assess.pred(as.numeric(APC.pred),unlist(valid.case))
APC.accuracy

#-------------------------  2. BAPC model     -----------------------------------
#
library(BAPC)
BAPC.case <- input.case
BAPC.pop <- pop[1:(nrow(input.case)+5),]
if(nrow(BAPC.case) != nrow(BAPC.pop)){
  diffrow <- nrow(BAPC.pop)-nrow(BAPC.case)
  add.dt <- data.frame(matrix(NA,nrow = diffrow,ncol=ncol(BAPC.case)))
  colnames(add.dt) <- colnames(BAPC.case)
  BAPC.case <- rbind(BAPC.case,add.dt)
}
bapc.list <- APCList(BAPC.case, BAPC.pop, gf = 5) #gf:age group interval
bapc.model <- BAPC(
  bapc.list,
  predict = list(npredict = 5, retro = FALSE),
  model = list(age = list(model = "rw2", prior="loggamma", param = c(1,
0.00005), initial = 4, scale.model=FALSE),
    period = list(include = TRUE, model = "rw2", prior="loggamma", param = c(1,
0.00005), initial = 4, scale.model=FALSE),
```

```
    cohort = list(include = TRUE, model = "rw2", prior="loggamma", param = c(1,
0.00005), initial = 4, scale.model=FALSE),
    overdis = list(include = TRUE, model = "iid", prior="loggamma", param = c(1,
0.005), initial = 4)),
    secondDiff = FALSE,
  #stdweight = weight,   #only for age-standardised projections
  verbose = FALSE
)
bapc.res <- agespec.proj(qapc(bapc.model, percentiles=c(0.025, 0.5, 0.975)))
bapc.predcase <- NULL
for(i in 1:ncol(case)){
  bapc.predcase <- cbind(bapc.predcase,tail(bapc.res[[i]][,4],n=diffrow))#4 for
median
}
bapc.accuracy <- assess.pred(as.vector(bapc.predcase),unlist(valid.case))


#-------------------------  3 Poisson model    ----------------------------------
-#
Poi.case <- data.frame(input.case)
Poi.case.true <-  data.frame(case[1:30,])
Poi.pop <- data.frame(pop[1:30,])
if(nrow(Poi.case) != nrow(Poi.pop)){
  diffrow <- nrow(Poi.pop)-nrow(Poi.case)
  add.dt <- data.frame(matrix(NA,nrow = diffrow,ncol=ncol(Poi.case)))
  colnames(add.dt) <- colnames(Poi.case)
  Poi.case <- rbind(Poi.case,add.dt)
}

#add year
Poi.case$year <- rownames(pop)[1:nrow(Poi.case)]
Poi.pop$year <- rownames(pop)[1:nrow(Poi.pop)]
Poi.case.true$year <- rownames(pop)[1:nrow(Poi.case.true)]
#transform wide to long
library(tidyr)
colnames(Poi.case.true)
Poi.case.true.long <- gather(Poi.case.true, key=agegroup, value=truecnt,
Age1:Age17)
Poi.case.long <- gather(Poi.case, key=agegroup, value=casecnt, Age1:Age17)
Poi.pop.long <- gather(Poi.pop, key=agegroup, value=popcnt, Age1:Age17)
Poi.data <- cbind(Poi.case.long,Poi.pop.long$popcnt,Poi.case.true.long$truecnt)
Poi.data$agegroup <- factor(Poi.data$agegroup)
Poi.data$year <- as.numeric(Poi.data$year)
colnames(Poi.data) <- c('year','agegroup','casecnt','popcnt','truecnt')

Poi.model <-
  glm(casecnt ~ year + agegroup , offset=log(popcnt),
      data = Poi.data,
      family = 'poisson')
Poi.pred <- predict(Poi.model,newdata = Poi.data,type='response')
Poi.accuracy <- assess.pred(Poi.pred[is.na(Poi.data$casecnt)],
                            Poi.data$truecnt[is.na(Poi.data$casecnt)])
Poi.accuracy

#-------------------------  4 negative binomial model    -------------------------
--#
require(MASS)
NB.model <-
```

```r
  glm.nb(casecnt ~ year + agegroup + offset(log(popcnt)),
         data = Poi.data)

NB.pred <- predict(NB.model,newdata = Poi.data,type='response')
NB.accuracy <- assess.pred(NB.pred[is.na(Poi.data$casecnt)],
                           Poi.data$truecnt[is.na(Poi.data$casecnt)])

#-------------------------  5 GAM    --------------------------#
library(mgcv)
Gam.model <- gam(casecnt ~ s(year) + agegroup+ offset(log(popcnt)),
                 data=Poi.data,
                 family=poisson(link="log"),method = "REML")
Gam.pred <- predict(Gam.model,newdata = Poi.data,type='response')
Gam.accuracy <- assess.pred(Gam.pred[is.na(Poi.data$casecnt)],
                            Poi.data$truecnt[is.na(Poi.data$casecnt)])
Gam.accuracy



#--------------------------FINAL OUTPTUT ------------------------------------#
accuracy.list <- c(Poi.accuracy,NB.accuracy,
                   SP.accuracy,Gam.accuracy,
                   APC.accuracy,bapc.accuracy)
names(accuracy.list) <- c('Poi.accuracy','NB.accuracy',
                          'SP.accuracy','Gam.accuracy',
                          'APC.accuracy','bapc.accuracy')
write.table(t(accuracy.list),'clipboard',col.names = F,row.names=F,sep='\t')
```
