# Supplementary material for: Secular trends of morbidity and mortality of prostate, bladder, and kidney cancers in China, 1990 to 2019 and their predictions to 2030
Source: BMC Cancer. 2022 Nov 11;22:1164. doi: 10.1186/s12885-022-10244-9 (PMC9650664; doi:10.1186/s12885-022-10244-9)
Supplement: Supplementary file 2 — Additional file 2. Mean absolute percentage error of 5 prediction models [file 12885_2022_10244_MOESM2_ESM.docx]

**Table Mean absolute percentage error of 5 prediction models.**

| Cancer type | Metric | Group | Poisson | NB | GAM | APC | BAPC |
| --- | --- | --- | --- | --- | --- | --- | --- |
| Overall (average) | | | 0.29 | 0.28 | 0.18 | 0.29 | **0.14** |
| Bladder cancer | Incidence | Total | 0.11 | 0.08 | 0.08 | 0.23 | 0.09 |
|  |  | Male | 0.13 | 0.11 | 0.09 | 0.24 | 0.11 |
|  |  | Female | 0.07 | 0.06 | 0.07 | 0.21 | 0.06 |
|  | Mortality | Total | 0.21 | 0.14 | 0.17 | 0.34 | 0.10 |
|  |  | Male | 0.19 | 0.13 | 0.16 | 0.28 | 0.11 |
|  |  | Female | 0.27 | 0.20 | 0.24 | 0.48 | 0.08 |
| Kidney cancer | Incidence | Total | 0.32 | 0.34 | 0.15 | 0.25 | 0.08 |
|  |  | Male | 0.39 | 0.39 | 0.16 | 0.28 | 0.13 |
|  |  | Female | 0.21 | 0.26 | 0.13 | 0.20 | 0.06 |
|  | Mortality | Total | 0.45 | 0.46 | 0.30 | 0.32 | 0.25 |
|  |  | Male | 0.55 | 0.54 | 0.31 | 0.32 | 0.31 |
|  |  | Female | 0.31 | 0.32 | 0.26 | 0.31 | 0.21 |
| Prostate cancer | Incidence | Male | 0.36 | 0.39 | 0.12 | 0.29 | 0.11 |
|  | Mortality | Male | 0.47 | 0.52 | 0.27 | 0.35 | 0.33 |

NB, negative binomial model; GAM, generalized additive model; APC, age-period-cohort study; BAPC, Bayesian age-period-cohort study.

Mean absolute percentage error, $\frac{1}{n}\times\sum\frac{|\hat{Y}-Y|}{Y}$, where Y and $\hat{Y}$denoted observed case and predicted case, respectively
