# Supplementary figures and images for: Secular trends of morbidity and mortality of prostate, bladder, and kidney cancers in China, 1990 to 2019 and their predictions to 2030
Source: BMC Cancer. 2022 Nov 11;22:1164. doi: 10.1186/s12885-022-10244-9 (PMC9650664; doi:10.1186/s12885-022-10244-9)

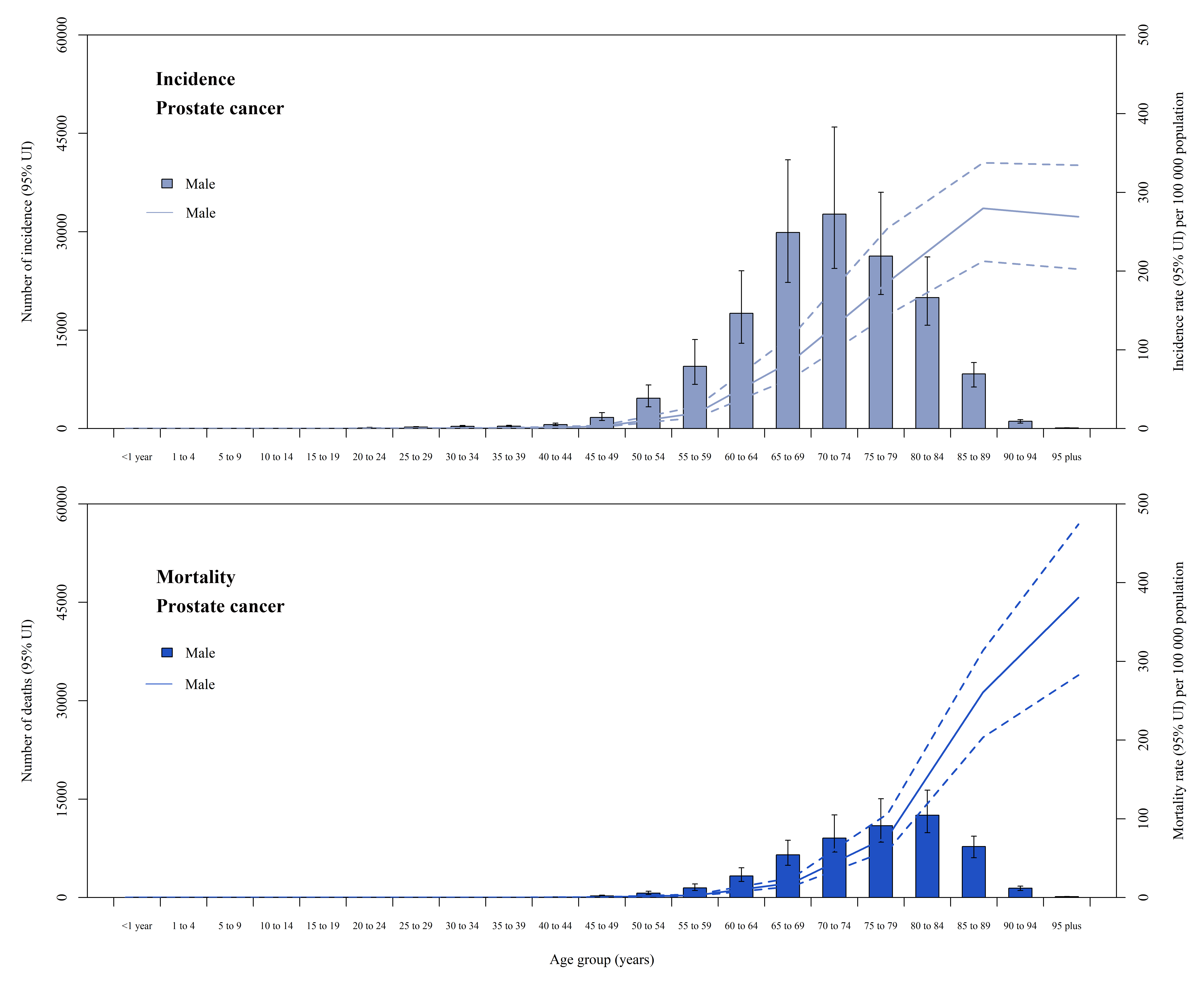

Supplement: Supplementary file 3 — Additional file 3. Figure S1 prostate cancer (1990-2019) [file 12885_2022_10244_MOESM3_ESM.tif]

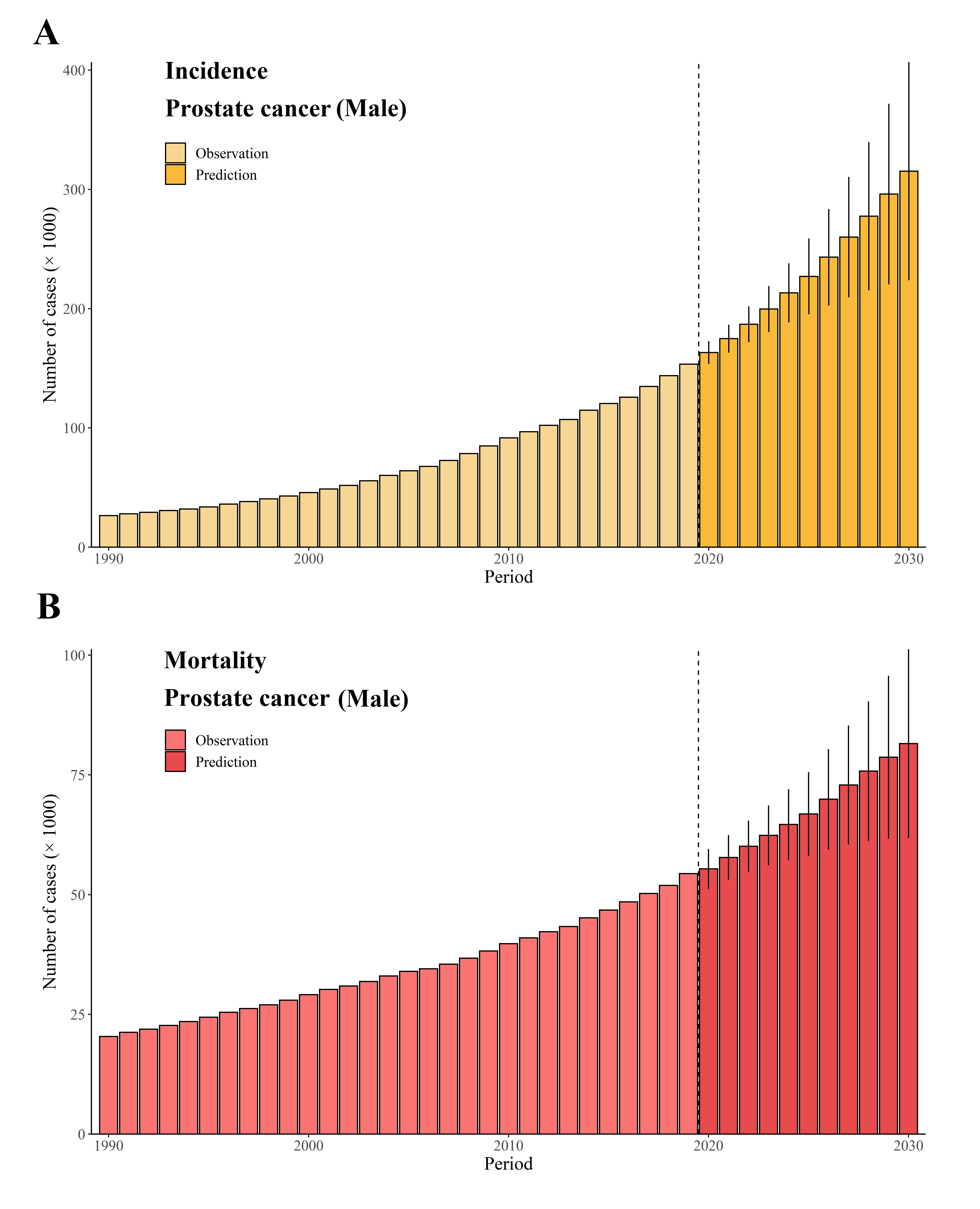

Supplement: Supplementary file 4 — Additional file 4. Figure S2 prostate cancer, projection of cases and deaths to 2030 [file 12885_2022_10244_MOESM4_ESM.tif]

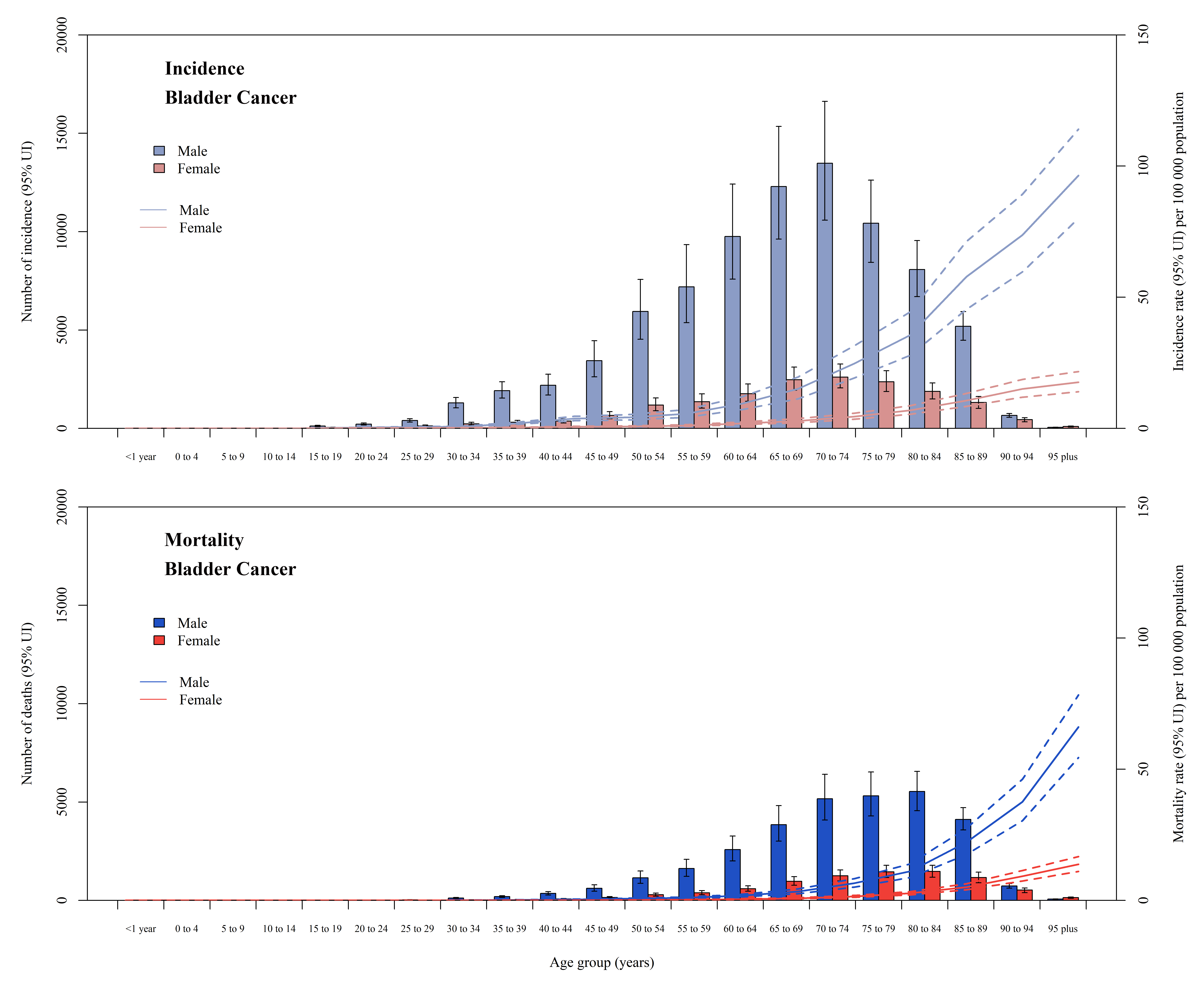

Supplement: Supplementary file 5 — Additional file 5. Figure S3 bladder cancer (1990-2019) [file 12885_2022_10244_MOESM5_ESM.tif]

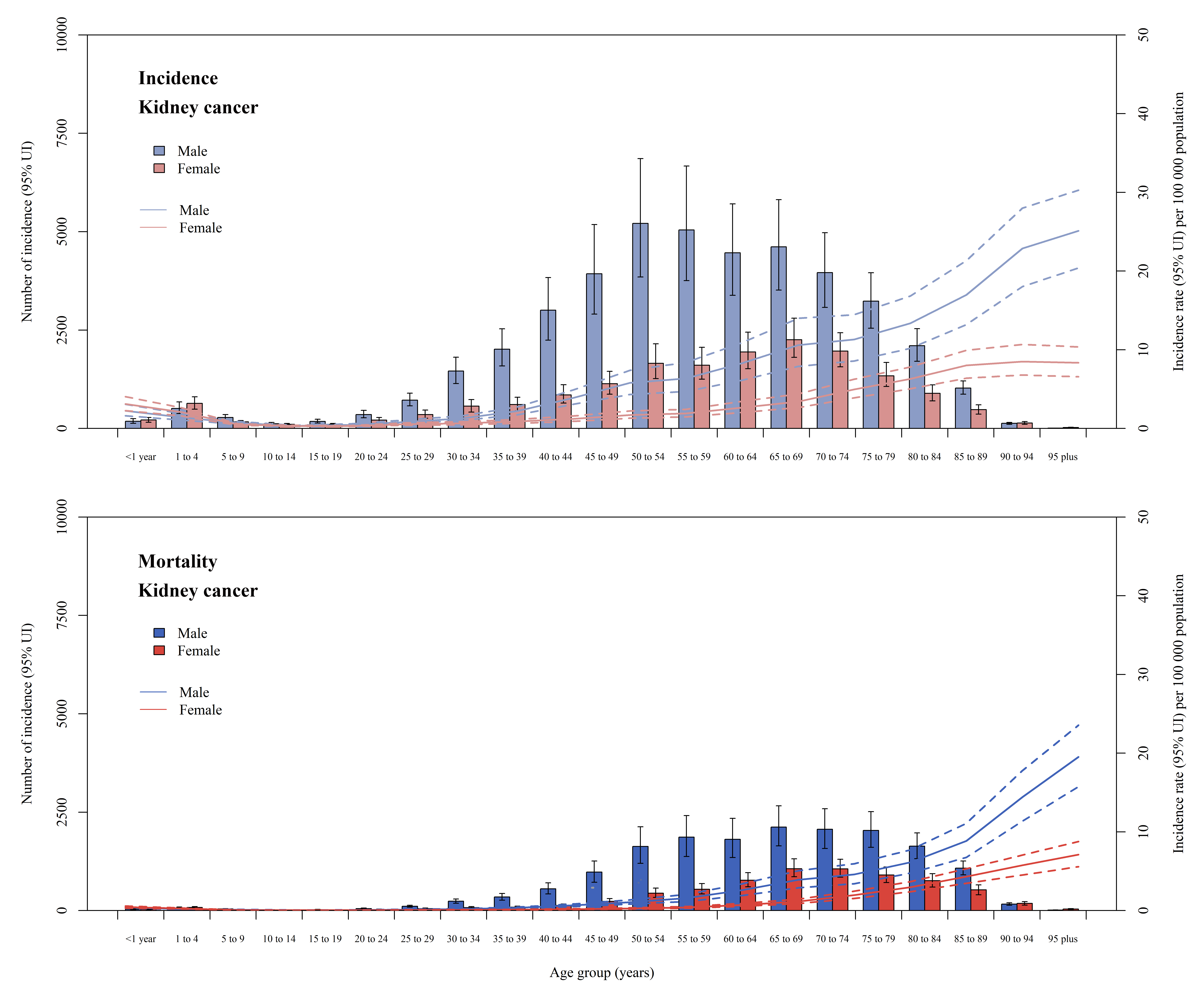

Supplement: Supplementary file 7 — Additional file 7. Figure S5 kidney cancer (1990-2019) [file 12885_2022_10244_MOESM7_ESM.tif]
